# CXCL1, RANTES, IFN-γ, and TMAO as Differential Biomarkers Associated with Cognitive Change After an Anti-Inflammatory Diet in Children with ASD and Neurotypical Peers

**DOI:** 10.3390/medsci14010011

**Published:** 2025-12-26

**Authors:** Luisa Fernanda Méndez-Ramírez, Miguel Andrés Meñaca-Puentes, Luisa Matilde Salamanca-Duque, Marysol Valencia-Buitrago, Andrés Felipe Ruiz-Pulecio, Carlos Alberto Ruiz-Villa, Diana María Trejos-Gallego, Juan Carlos Carmona-Hernández, Sandra Bibiana Campuzano-Castro, Marcela Orjuela-Rodríguez, Vanessa Martínez-Díaz, Jessica Triviño-Valencia, Carlos Andrés Naranjo-Galvis

**Affiliations:** 1 Facultad de Estudios Sociales y Empresariales, Doctorado en Ciencias Cognitivas, Universidad Autónoma de Manizales, Antigua Estación del Ferrocarril, Manizales 170004, Colombia; 2 Facultad de Inteligencia Artificial e Ingeniería, Universidad de Caldas, Manizales 170004, Colombia; 3 Facultad de Salud, Universidad Autónoma de Manizales, Antigua Estación del Ferrocarril, Manizales 170004, Colombia; 4 Facultad de Ciencias de la Salud, Universidad Católica de Manizales, Manizales 170004, Colombia; 5 Facultad de Ciencias de la Salud, Universidad de Manizales, Manizales 170004, Colombia; 6 Grupo de Investigación Médica, Universidad de Manizales, Manizales 170004, Colombia; 7 Universidad Católica Luis Amigó, Sede Manizales, Manizales 170004, Colombia; 8 Centro de Bioinformática y Biología Computacional de Colombia (BIOS), Manizales 170004, Colombia; 9 Grupo de Investigación Biomedicina, Institución Universitaria Visión de las Américas, Pereira 660003, Colombia

**Keywords:** autism spectrum disorder, anti-inflammatory diet, neuroimmune modulation, TMAO, cytokines, immune–cognitive networks

## Abstract

**Background/Objective**: Neuroimmune and metabolic dysregulation have been increasingly implicated in the cognitive heterogeneity of autism spectrum disorder (ASD). However, it remains unclear whether anti-inflammatory diets engage distinct biological and cognitive pathways in autistic and neurotypical children. This study examined whether a 12-week anti-inflammatory dietary protocol produces group-specific neuroimmune–metabolic signatures and cognitive responses in autistic children, neurotypical children receiving the same diet, and untreated neurotypical controls. **Methods**: Twenty-two children (11 with ASD, six a on neurotypical diet [NT-diet], and five neurotypical controls [NT-control]) completed pre–post assessments of plasma IFN-γ, CXCL1, RANTES (CCL5), trimethylamine-N-oxide (TMAO), and an extensive ENI-2/WISC-IV neuropsychological battery. Linear mixed-effects models were used to test the Time × Group effects on biomarkers and cognitive domains, adjusting for age, sex, and baseline TMAO. Bayesian estimation quantified individual changes (posterior means, 95% credible intervals, and posterior probabilities). Immune–cognitive coupling was explored using Δ–Δ correlation matrices, network metrics (node strength, degree centrality), exploratory mediation models, and responder (≥0.5 SD domain improvement) versus non-responder analyses. **Results**: In ASD, the diet induced robust reductions in IFN-γ, RANTES, CXCL1, and TMAO, with decisive Bayesian evidence for IFN-γ and RANTES suppression (posterior P(δ < 0) > 0.99). These shifts were selectively associated with gains in verbal learning, semantic fluency, verbal reasoning, attention, and visuoconstructive abilities, whereas working memory and executive flexibility changes were heterogeneous, revealing executive vulnerability in individuals with smaller TMAO reductions. NT-diet children showed modest but consistent improvements in visuospatial processing, attention, and processing speed, with minimal biomarker changes; NT controls remained biologically and cognitively stable. Network analyses in ASD revealed a dense chemokine-anchored architecture with CXCL1 and RANTES as central hubs linking biomarker reductions to improvements in fluency, memory, attention, and executive flexibility. ΔTMAO predicted changes in executive flexibility only in ASD (explaining >50% of the variance), functioning as a metabolic node of executive susceptibility. Responders displayed larger coordinated decreases in all biomarkers and broader cognitive gains compared to non-responders. **Conclusions**: A structured anti-inflammatory diet elicits an ASD-specific, coordinated neuroimmune–metabolic response in which suppression of CXCL1 and RANTES and modulation of TMAO are tightly coupled with selective improvements in verbal, attentional, and executive domains. Neurotypical children exhibit modest metabolism-linked cognitive benefits and minimal immune modulation. These findings support a precision-nutrition framework in ASD, emphasizing baseline immunometabolic profiling and network-level biomarkers (CXCL1, RANTES, TMAO) to stratify responders and design combinatorial interventions targeting neuroimmune–metabolic pathways.

## 1. Introduction

Autism Spectrum Disorder (ASD) is a heterogeneous neurodevelopmental condition characterized by impairments in social communication, restricted and repetitive behaviors, and marked inter-individual variability across cognitive, sensory, and emotional domains [1]. Contemporary neuropsychological research indicates that ASD encompasses diverse cognitive phenotypes, frequently involving alterations in attention, working memory, executive functioning, and language-related processes that evolve across developmental stages [2,3,4]. Globally, the prevalence of ASD has increased over the past decade, with substantial regional variability. Recent estimates from Latin America range from 0.28% to 16%, reflecting disparities in diagnostic access, early surveillance, and epidemiological reporting systems [5,6]. Early behavioral markers, such as diminished eye contact, impaired joint attention (defined as difficulties in coordinating attention between a social partner and an object or event), reduced imitation, and delayed response to names, remain among the most reliable predictors of ASD during the first two years of life [7].

Beyond behavioral manifestations, converging evidence supports immune dysregulation as the core component of ASD neurobiology. Meta-analyses and large cohort studies have consistently reported elevated levels of pro-inflammatory cytokines and chemokines, such as IFN-γ, IL-6, IL-17A, CXCL8, and CCL5/RANTES, together with altered microglial activation patterns, which have been linked to executive dysfunction, irritability, and behavioral inflexibility [8,9,10,11]. In parallel, gastrointestinal disturbances, including constipation, diarrhea, and abdominal pain, are highly prevalent in ASD and are strongly associated with emotional dysregulation and maladaptive behaviors, reinforcing the mechanistic relevance of the gut–immune–brain axis in shaping ASD symptomatology during childhood [12,13,14].

In this context, increasing attention has shifted beyond classical cytokines toward gut microbiota-derived metabolites as immunometabolic mediators of neurocognitive vulnerability. Among these, trimethylamine-N-oxide (TMAO), a hepatic oxidation product of microbial trimethylamine derived from dietary choline, carnitine, and phosphatidylcholine, has emerged as a biologically active effector linking diet, gut microbiota composition, immune activation, and brain function [15,16,17]. Accumulating experimental and clinical evidence indicates that elevated TMAO levels promote oxidative stress, endothelial dysfunction, mitochondrial impairment, and activation of the NLRP3 inflammasome, with downstream effects on systemic and central inflammatory signaling [18,19]. These processes may disrupt blood–brain barrier integrity, modulate microglial activation, and alter neuronal energy metabolism, thereby influencing attentional control, executive flexibility, and cognitive resilience [15,16,20]. Notably, children with autism spectrum disorder (ASD) appear particularly susceptible to microbiota-derived metabolic perturbations, with emerging studies linking elevated circulating TMAO to behavioral rigidity, executive dysfunction, and broader neurodevelopmental vulnerabilities [21,22].

Building on these mechanistic insights, recent synthesis studies conducted outside the ASD field have identified TMAO as a key immunometabolic mediator of cognitive vulnerability across diverse clinical populations. A systematic review and meta-analysis demonstrated consistent associations between circulating TMAO and its dietary precursors with cognitive impairment, executive dysfunction, and neurodegenerative risk, underscoring its role as an active biological effector rather than a passive metabolic byproduct of the gut microbiota [23]. Complementarily, meta-analytic evidence indicates that dietary modulation through prebiotics and phytochemicals can significantly reduce serum TMAO levels while reshaping the gut microbial composition, highlighting the modifiability of this pathway through nutritional interventions [24]. Advances in immunometabolism and systems immunology have further demonstrated that TMAO-driven metabolic stress intersects with inflammatory signaling, oxidative stress, endothelial dysfunction, and inflammasome activation, mechanisms that directly influence brain function and executive control. Despite this robust body of evidence, TMAO has rarely been incorporated into multidimensional models integrating immune signaling and domain-specific cognitive outcomes in ASD, leaving its role as a metabolic node underlying inter-individual variability in cognitive responsiveness largely unexplored [18,19,20,25].

Therefore, nutritional interventions have been proposed as complementary strategies to modulate systemic inflammation, gut microbiota composition, and neurocognitive performance. Anti-inflammatory dietary patterns rich in omega-3 fatty acids, polyphenols, antioxidants, and fermentable fibers (i.e., microbiota-accessible carbohydrates such as inulin, fructooligosaccharides, resistant starch, and β-glucans, which are metabolized by gut bacteria) have demonstrated benefits in psychiatric and neurological populations, including improvements in attention, cognitive flexibility, and mood regulation [26,27,28]. However, evidence supporting diet-related cognitive improvements in ASD remains limited due to small sample sizes, lack of neurotypical comparison groups, minimal integration of immunometabolic markers, and the absence of analytical approaches capable of capturing coordinated biological interactions. Preliminary findings from Colombian cohorts suggest that anti-inflammatory diets may reduce cytokine levels in children with ASD, although the cognitive pathways underlying these effects remain poorly understood [29,30,31].

Emerging integrative reviews emphasize the central role of gut microbiota alterations, immune dysregulation, and metabolic imbalance in shaping neurodevelopmental outcomes; however, these components are most often examined in isolation rather than as coordinated systems [32]. Consequently, critical gaps remain in the literature. Specifically, microbiota-derived metabolites such as TMAO have rarely been analyzed alongside inflammatory chemokines and cognitive outcomes within a unified framework, and systems-level approaches capable of capturing coordinated neuroimmune–metabolic adaptation are scarce in ASD nutritional research. This limitation hampers the ability to explain the interindividual variability in dietary responsiveness and cognitive plasticity.

To address these gaps, the present study examined neuropsychological performance, cytokine profiles, and TMAO metabolism in autistic children (*n* = 11), neurotypical children receiving the same anti-inflammatory diet (*n* = 6), and neurotypical controls without intervention (*n* = 5). By integrating linear mixed-effects modeling, Bayesian estimation of individual change, immune–cognitive network analysis, and responder stratification, this study moves beyond group-average effects to identify coordinated neuroimmune–metabolic signatures associated with selective cognitive gains. This systems-level framework provides a mechanistically grounded basis for precision-nutrition approaches in ASD, emphasizing biological heterogeneity and individualized pathways of cognitive responsiveness rather than one-size-fits-all dietary interventions.

## 2. Materials and Methods

### 2.1. Study Design

This investigation employed a 12-week quasi-experimental pre–post intervention design in which all children with autism spectrum disorder (ASD) received an anti-inflammatory dietary protocol (*NeuroGutPlus*), while neurotypical (NT) children were assigned either to the same dietary intervention or to a non-intervention comparison group to evaluate cognitive, immunological, and metabolic outcomes.

The design included two main groups: children with Autism Spectrum Disorder (ASD) and neurotypical participants drawn from the same geographic region. All children with autism spectrum disorder (ASD) were assigned to the dietary intervention group, as withholding nutritional intervention was considered ethically inappropriate given caregiver interest and the exploratory nature of the study. Neurotypical participants were allocated to either the dietary intervention group or a non-intervention control group based on caregiver consent, feasibility of adherence to the dietary protocol, and scheduling availability. Throughout the manuscript, the term “NT-Control” is used to denote neurotypical participants who did not receive the dietary intervention and served as a non-intervention comparison group. This group represented an observational reference condition characterized by cognitive and biological stability over the study period rather than a randomized experimental control. Therefore, allocation was pragmatic rather than randomized, and no participants were excluded based on group assignment. This allocation strategy was selected to maximize the feasibility and ecological validity in a real-world pediatric setting. Consequently, analytical approaches emphasizing within-subject change, mixed-effects modeling, and Bayesian inference were employed to appropriately account for the non-randomization and small sample size.

The study groups were not formally matched a priori for age, sex, baseline cognitive scores, or socioeconomic status due to the exploratory and feasibility-oriented nature of the study and the modest sample size. Therefore, baseline comparability across groups was assessed empirically. Age and sex distributions were summarized descriptively, and baseline neuropsychological performance was evaluated prior to the intervention. No statistically significant differences were observed between the groups in the primary cognitive domains at baseline.

Socioeconomic status was not used as a matching criterion; however, all participants were recruited from the same geographic region and educational setting, which reduced the large socioeconomic variability. To further address potential confounding, age and sex were included as covariates in the statistical models, and analyses emphasized the within-subject change over time.

**Figure 1:**
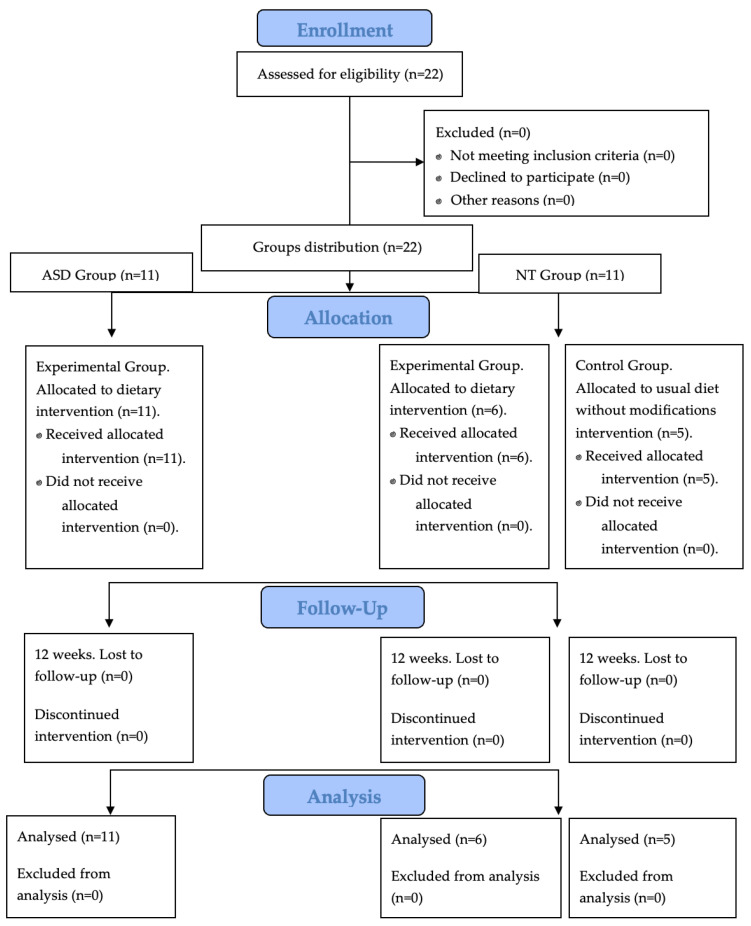
Flow diagram of the study design showing participant enrollment, group allocation (ASD, neurotypical diet, and neurotypical control), follow-up over 12 weeks, and inclusion in the final analysis. No participants were lost to follow-up or excluded from the analyses.

All procedures adhered to the ethical principles of the Declaration of Helsinki and the Colombian Regulatory Framework. The flow of participants through the study, including enrollment, group allocation, follow-up, and analysis, is illustrated in Figure 1.

A total of 22 children aged 6–16 years completed the study: 11 diagnosed with ASD and 11 who were neurotypical controls. ASD diagnoses were confirmed by trained clinicians using the DSM-5 criteria and standardized developmental histories. Participants were recruited from Manizales, Colombia. Children with ASD were identified through local support institutions, and NT participants were recruited from the same communities to ensure comparable socio-demographic and environmental characteristics. The inclusion criteria required active enrollment in the Colombian General Social Security Health System (SGSSS), absence of recent (past 3 months) corticosteroid, antifungal, antibiotic, or immunosuppressive use, no special diet at baseline, and absence of food intolerances or allergy to milk proteins. Children were excluded if they presented with neurological, neuromuscular, psychiatric, metabolic, systemic inflammatory, gastrointestinal, or immunological disorders, including celiac disease. Exclusion criteria were assessed at baseline through a structured caregiver interview conducted by trained study personnel, supplemented by a review of the available medical and developmental records. Information on neurological and psychiatric comorbidities, chronic inflammatory or autoimmune conditions, active infections, gastrointestinal diseases requiring medical treatment, recent antibiotic or immunomodulatory use, and special dietary regimens was collected using standardized health questionnaires that were completed by caregivers. When applicable, prior diagnoses were verified using medical documentation provided by the family. Only participants who met all the inclusion criteria and none of the exclusion criteria were enrolled.

Written informed consent was obtained from all parents or legal guardians, and all evaluations were conducted individually at the Universidad Autónoma de Manizales. The study was conducted in accordance with the Declaration of Helsinki and approved by the Committee on Ethics, Bioethics, and Scientific Integrity of the Universidad Autónoma de Manizales (Acta No. 150, Approval Date: 10 May 2023). Informed consent was obtained from all the participants involved in the study. The study was registered in the International Standard Randomised Controlled Trial Number (ISRCTN) registry under the registration number ISRCTN15439377 (DOI: 10.1186/ISRCTN15439377). The registration date was 24 September 2025. The study was retrospectively registered after completion, as data collection and follow-up were concluded prior to registry submission.

Owing to the nature of the dietary intervention, participant and caregiver blinding was not feasible, and expectancy or placebo effects—particularly in cognitive outcomes—could not be fully excluded. To mitigate this risk, the study design included two neurotypical comparison groups (dietary intervention and non-intervention control), enabling differentiation between general expectancy-related changes and ASD-specific neuroimmune and metabolic responses. In addition, analyses emphasized within-subject pre–post changes and integrated cognitive outcomes with objective biological measures, thereby reducing reliance on subjective improvement alone.

### 2.2. Dietary Intervention: NeuroGutPlus

*NeuroGutPlus* is a structured anti-inflammatory nutritional intervention developed to promote gut barrier integrity and reduce systemic inflammation. The 12-week protocol restricted gluten (proteins found in wheat, barley, and rye), fermentable carbohydrates (FODMAPs; fermentable oligosaccharides, disaccharides, monosaccharides, and polyols, such as fructans in wheat and onions, lactose in dairy products, excess fructose in certain fruits, and polyols in sweeteners), casein (the main milk protein present in dairy products such as cow’s milk, cheese, and yogurt), ultra-processed foods (including packaged snacks, processed meats, and ready-to-eat meals), and artificial additives (such as colorants, preservatives, and flavor enhancers).

Concurrently, the dietary protocol increased the intake of omega-3 fatty acids (e.g., fatty fish such as salmon and sardines), polyphenols (e.g., olive oil and cocoa), high-fiber foods (e.g., vegetables, legumes, and whole foods), and fermentable prebiotics (e.g., inulin and resistant starch) to support gut microbial modulation and anti-inflammatory effects.

Families received standardized food packages and participated in training sessions to ensure the correct preparation and administration of meals. Quantitative nutrient intake estimates (e.g., grams per day, caloric intake, or macronutrient distribution) were not formally calculated in this study. The dietary intervention was implemented using a structured, food-based anti-inflammatory protocol that emphasized food groups and dietary patterns rather than precise nutrient quantification. This approach was selected to enhance the feasibility, caregiver adherence, and ecological validity in a pediatric population. Given these design characteristics, analytical strategies emphasized within-subject pre–post comparisons and the inclusion of neurotypical dietary and nondietary comparison groups to contextualize observed biological and cognitive changes.

Adherence to the dietary protocol was evaluated using a multimodal strategy. Caregivers completed weekly dietary records documenting the consumption of permitted and restricted food items. A nutritionist conducted weekly telephone follow-ups to address the challenges and reinforce the guidelines. Additionally, the remaining food items from the weekly packages were verified during the scheduled check-ins. These measures consistently indicated dietary adherence exceeding 85%, supporting the fidelity of the intervention. Adherence was operationalized as the proportion of predefined dietary recommendations and restrictions fulfilled over the 12-week intervention period, as documented through weekly caregiver-completed food diaries and structured dietary checklists. These instruments captured compliance with the key components of the *NeuroGutPlus* protocol, including avoidance of restricted food categories and inclusion of prescribed food groups. Accordingly, an adherence rate of 85% reflects compliance with approximately 85% of the specified dietary criteria across the intervention period, rather than food weight, calorie intake, or frequency of consumption.

In contrast, participants in the neurotypical control group maintained their regular habitual diet throughout the 12-week period and did not receive any dietary modifications or structured nutritional guidance. This distinction preserved the natural dietary patterns of the control group and strengthened the comparative evaluation of the effects of the intervention.

### 2.3. Sociodemographic, Clinical, and Nutritional Assessment

At baseline, caregivers completed a sociodemographic and clinical questionnaire that documented age, sex, socioeconomic status, school level, medical history, and the presence of pediatric gastrointestinal symptoms. Nutritional status was assessed in all participants by a trained nutritionist using standardized measurements of weight, height, and body mass index (BMI). Classifications were performed using the anthropometric criteria established by Resolution 2465 of 2016 (Ministry of Health and Social Protection, Bogotá, Colombia). These assessments informed individualized caloric distribution and macronutrient planning within the dietary interventions.

### 2.4. Neuropsychological Assessment

Neuropsychological evaluation was conducted at baseline and after the 12-week intervention period. It included the administration of the Wechsler Intelligence Scale for Children—Fourth Edition (WISC-IV) and selected subtests from the Neuropsychological Evaluation of Children—Second Edition (ENI-2). The WISC-IV provides a Full-Scale IQ score and four primary indices—Verbal Comprehension, Perceptual Reasoning, Working Memory, and Processing Speed—derived from ten core subtests. Index scores were reported as standardized scores (mean = 100, SD = 15), and subtests were reported as scaled scores (mean = 10, SD = 3). The ENI-2 assessment focused on cognitively sensitive subtests, including Stick Construction, Human Figure, Complex Figure copy and recall, Word List Learning and its associated recall trials, Auditory Verbal Recognition, Instruction Following, Picture Cancellation, Letter Cancellation, Semantic Fluency (fruits and animals), Phonemic Fluency, and Cognitive Flexibility (CF). These tasks evaluate visuoconstruction, memory, attention, verbal learning, and executive functioning.

### 2.5. Biomarker Collection and Quantification

Blood samples were obtained at baseline and week 12 under standardized morning conditions. Plasma was separated by centrifugation (1500× *g*, 10 min) and immediately aliquoted and stored at −80 °C until analysis to prevent analyte degradation. All inflammatory and metabolic biomarkers were quantified using validated, commercially available ELISA kits (Thermo Fisher Scientific, Waltham, MA, USA), following the manufacturer’s protocols and established guidelines for immunoassay-based cytokine quantification.

Plasma cytokines (IFN-γ, CXCL1, and RANTES/CCL5) were measured using human-specific sandwich ELISA kits supplied by Abcam (Cambridge, UK). The following kits were used: Human IFN-γ ELISA Kit (catalog no. ab174443), and Human CXCL1 ELISA Kit (catalog no. ab190805) and Human RANTES/CCL5 ELISA Kit (catalog no. ab174446). All samples and standards were assayed in duplicate, and concentration values were derived from 4-parameter logistic calibration curves with internal controls included on every plate. Measurements below the lower limit of detection (LOD) were treated using the LOD/√2 substitution method.

Plasma trimethylamine-N-oxide (TMAO) levels were quantified using a specific human ELISA assay (AFG Bioscience, Northbrook, IL, USA; catalog no. EK715704). Absorbance was recorded at 450 nm, and quantification was performed using standard curves and the manufacturer’s quality control materials. As with cytokines, all samples were analyzed in duplicate to reduce analytical variability.

The analytical performance met the established quality criteria for ELISA-based biomarker assessment. The intra-assay coefficients of variation remained below 10%, and the inter-assay variability was consistently below 15% for all analytes. This level of precision, combined with duplicate measurements, curve-based validation, and standardized QC procedures, supports the reliability and robustness of the biomarker dataset used in subsequent statistical modeling, including mixed-effects analyses, Bayesian inference, and network-based modeling.

### 2.6. Statistical Analysis

Primary analyses employed linear mixed-effects models (LMMs) to evaluate the effects of time (pre vs. post) and group (ASD vs. NT) on cytokines, TMAO, neuropsychological subtests, and composite domains. Each model included fixed effects for Time, Group, and baseline TMAO, with a random intercept for each participant to account for repeated measures. Bayesian estimation procedures were applied to quantify individual change trajectories using weakly informative priors to generate posterior means, 95% credible intervals, and posterior probabilities of improvement or deterioration. Immune–cognitive network analyses were performed using Spearman correlation matrices of within-subject change scores (Δ), retaining edges with |ρ| ≥ 0.50 and *p* < 0.05. Network metrics, including node strength and degree centrality, were calculated to identify potential mechanistic hubs. Responders were defined as participants who exhibited either an increase of ≥0.5 SD in cognitive domain scores or a decrease of ≥0.5 SD in cytokine concentrations.

Given the simultaneous analysis of multiple cytokines and chemokines, the risk of false-positive findings was addressed using a false discovery rate (FDR) correction based on the Benjamini–Hochberg procedure. FDR-adjusted q-values were calculated for cytokine-related analyses involving multiple parallel tests, and statistical significance was interpreted primarily based on adjusted results rather than uncorrected *p*-values. In addition to FDR correction, effect sizes, consistency of directionality across analytical approaches, and biological coherence within immune–cognitive networks were considered when interpreting cytokine findings. Linear mixed-effects models adjusted for age and sex were used to reduce spurious associations related to demographic variability. This layered analytical strategy was selected to balance statistical stringency with sensitivity in the context of a small-sample, exploratory study.

Logistic regression models were used to evaluate the influence of baseline cytokines, TMAO, and cognitive scores on the responder status. To assess metabolic contributions, regression models were used to examine ΔTMAO as a predictor of Δexecutive functioning performance, including interaction terms to evaluate ASD-specific susceptibility. False discovery rate correction (Benjamini–Hochberg) was applied to the subtest-level analyses. All analyses were conducted using R (version 4.3.2; packages *lme4* version 1.1-35, *brms* version 2.21.0, and *qgraph* version 1.9.5; R Foundation for Statistical Computing, Vienna, Austria), Python (version 3.11; libraries *statsmodels* version 0.14.1 and *SciPy* version 1.11.4), GraphPad Prism (version 10.2.0; GraphPad Software, San Diego, CA, USA), and JASP (version 0.18.3; JASP Team, Amsterdam, The Netherlands).

## 3. Results

### 3.1. Baseline Characteristics

Prior to the longitudinal analyses, baseline demographic, cognitive, and biomarker characteristics were descriptively examined across groups. No marked differences were observed in the age or sex distribution. As expected, autistic children exhibited lower baseline performance in selected cognitive domains than neurotypical peers. Baseline cytokine concentrations and TMAO levels were comparable across the groups. These baseline observations provided the necessary context for interpreting the subsequent time × group effects.

### 3.2. Effects of the Anti-Inflammatory Diet on Cytokine and Metabolic Biomarkers

To ensure transparency in subtest-level analyses involving multiple simultaneous comparisons, both unadjusted *p*-values and false discovery rate (FDR)-adjusted q-values (Benjamini–Hochberg procedure) were calculated. Unless otherwise stated, the subtest-level findings described below refer to results that remained statistically significant after FDR correction. 

#### Linear Mixed-Effects Models (LMM)

**Figure 2:**
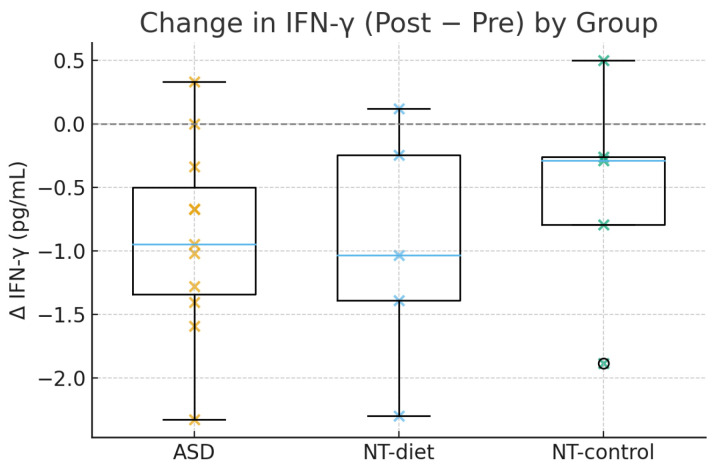
Changes in IFN-γ (Post–Pre) by group. Boxplots with individual data points showing the distribution of Δ IFN-γ (post − pre, pg/mL) in children with ASD, NT-diet, and NT-control. Negative values indicate a reduction after the intervention. Children with ASD showed larger and more consistent decreases compared with both neurotypical groups, in line with the significant Time × Group interaction detected in the LMM.

The anti-inflammatory diet elicited group-specific changes in inflammatory signaling, most prominently in IFN-γ. The linear mixed-effects model revealed a significant Time × Group interaction for IFN-γ (β_interaction < 0, *p* < 0.05), indicating that reductions over time were greater in children with ASD than in neurotypical participants. As illustrated in Figure 2, the distribution of individual change scores (ΔIFN-γ = post − pre) showed a clear shift in the ASD group compared with both NT-diet and NT-control children. Post hoc contrasts confirmed a marked decrease in IFN-γ among ASD participants (β = −0.42, 95% CI −0.70 to −0.15, *p* = 0.003), whereas neurotypical children exhibited minimal or no change (global NT *p* = 0.41). These results support the selective immunomodulatory effects of dietary interventions in children with ASD.

**Figure 3:**
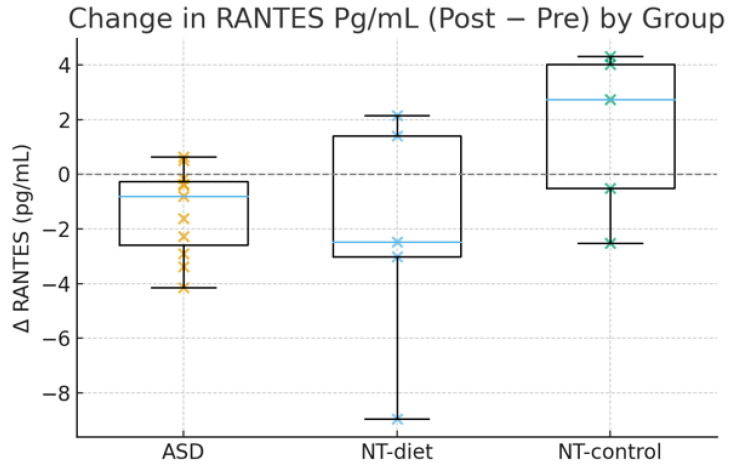
Group-wise distribution of post–pre changes (Δ) in RANTES (pg/mL). The ASD participants showed consistent reductions, whereas the NT diet and NT control groups displayed minimal or inconsistent changes. These results align with the LMM findings, showing a significant decrease in RANTES levels among ASD participants and a near-significant time × group interaction.

The anti-inflammatory diet produced a clear downward shift in RANTES concentrations in children with ASD, whereas changes in neurotypical participants were heterogeneous and did not follow a consistent direction. As shown in Figure 3, the ASD participants exhibited predominantly negative ΔRANTES values (post − pre), reflecting a reliable suppression of this chemokine. This aligns with the LMM results, indicating a robust and statistically significant reduction in ASD (β = −0.51, 95% CI −0.82 to −0.20, *p* = 0.002). In contrast, both the NT diet and NT control groups displayed wide variability, with mean changes close to zero (*p* = 0.27). Although the Time × group interaction did not reach conventional significance (*p* = 0.06), the visual and statistical trends strongly support differential immunological responsiveness, with ASD showing a more biologically coherent anti-inflammatory shift.

**Figure 4:**
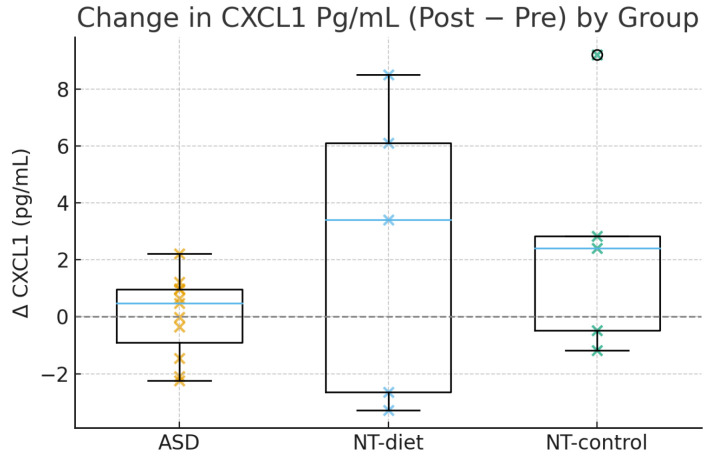
Post–pre change (Δ) in CXCL1 levels (pg/mL) across the ASD, NT diet, and NT control groups. Participants with ASD demonstrated a moderate but consistent reduction in CXCL1, whereas NT children presented heterogeneous responses. These changes parallel the significant LMM Time effect observed in ASD and the strong predictive association between ΔCXCL1 and cognitive improvement.

Changes in CXCL1 further reinforced the group-specific pattern of immunological response. As illustrated in Figure 4, children with ASD showed predominantly negative or near-zero ΔCXCL1 values, consistent with the LMM results that identified a moderate but significant decline (β = −0.31, *p* = 0.04). In contrast, the NT-diet participants exhibited a broader distribution with both positive and negative changes, suggesting higher biological noise and weaker responsiveness to the intervention. Interestingly, two NT-control subjects showed substantial positive increases in CXCL1, further supporting the notion that the observed CXCL1 reduction is specific to ASD and unlikely to represent a non-specific temporal effect. These findings are especially meaningful considering that CXCL1 reductions were strongly predictive of neurocognitive improvement, particularly in executive flexibility and attentional control.

**Figure 5:**
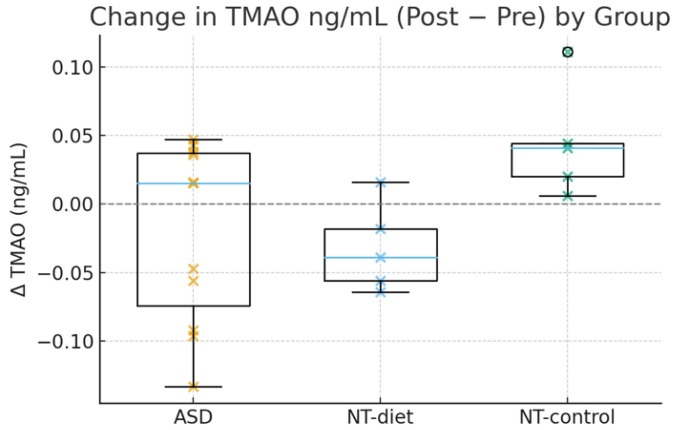
Group comparisons of ΔTMAO (ng/mL). The ASD participants exhibited clear reductions following the anti-inflammatory diet, while NT-diet children showed minimal changes, and NT-control participants showed increases. The LMM confirmed a significant metabolic shift in ASD, and ΔTMAO strongly predicted improvement in executive flexibility.

The metabolic responses to dietary interventions differed significantly between the groups. As shown in Figure 5, children with ASD demonstrated a clear tendency toward negative ΔTMAO values, indicating reductions after the anti-inflammatory diet. This pattern is consistent with the LMM analysis showing a significant metabolic shift among children with ASD (β = −0.46, 95% CI −0.80 to −0.10, *p* = 0.01).

In contrast, the NT diet group exhibited minimal change, with ΔTMAO values clustering near zero, whereas NT controls showed positive increases, suggesting progressive metabolic divergence in the absence of dietary modification. These effects highlight that the anti-inflammatory diet exerts a selective normalizing effect on TMAO metabolism that is unique to ASD.

Crucially, the Time × Group interaction was statistically significant (*p* < 0.05), and reductions in TMAO levels correlated strongly with improvements in executive flexibility among ASD participants. This suggests that TMAO may be an immunometabolic mediator linking dietary modulation to cognitive enhancement.

Together, these findings indicate that an anti-inflammatory diet exerts coordinated immunometabolic effects that are particularly salient in ASD and that these biological changes are meaningfully linked with cognitive outcomes.

### 3.3. Cognitive Outcomes

#### 3.3.1. Global Pre–Post Cognitive Effects Across Groups

Pre–post analyses revealed distinct cognitive trajectories across the three groups, which were consistent with the linear mixed-effects modeling results. In autistic children, the anti-inflammatory diet produced broad neurocognitive improvements across several ENI-2 and WISC-IV domains. Significant gains were observed in attentional efficiency and processing speed, particularly in Picture Cancellation, Letter Cancellation, and rapid visual scanning tasks (β range: 0.28–0.45, *p* < 0.05). Working memory performance also improved, most notably in the WISC-IV Digit Span and ENI-2 verbal recall measures (β ≈ 0.34, *p* = 0.02). Enhancements in visuoconstructive skills were also evident, with significant increases in Figure Copy and Block Design scores (β range: 0.22–0.30, *p* < 0.05). Effect sizes ranged from moderate to large (Cohen’s d = 0.45–0.92), with the strongest improvements in executive flexibility (d = 0.92), semantic fluency (d = 0.81), and Digit Span working memory (d = 0.77).

**Figure 6:**
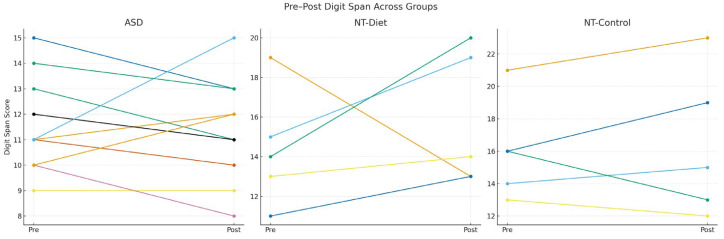
Pre–Post Digit Span Trajectories Across ASD, NT Diet, and NT Control Groups. Individual spaghetti plots displaying pre–post changes in Digit Span performance for the three study groups. Each line represents the trajectory of an individual child evaluated in the study, grouped according to diagnostic and intervention status. Children with Autism Spectrum Disorder (ASD) exhibited heterogeneous trajectories, with several participants showing meaningful post-intervention improvements, while others remained stable or demonstrated mild declines. Neurotypical children receiving an anti-inflammatory diet (NT-Diet) showed a more uniform upward trend, indicating consistent gains in working memory efficiency. In contrast, neurotypical controls without intervention (NT-Control) demonstrated minimal intra-individual variability and no systematic pre–post changes. These patterns visually complement the statistical findings, supporting the interpretation that working memory improvements in ASD and NT-Diet participants are attributable to the dietary intervention rather than practice or maturation effects.

These model-based findings are visually supported by the individual pre–post trajectories shown in Figure 6, which displays Digit Span performance across the ASD, NT-Diet, and NT-Control groups. In ASD, Digit Span trajectories were heterogeneous but demonstrated an overall upward trend following the intervention. Neurotypical children who received the diet showed a more uniform improvement pattern, with nearly all trajectories increasing from the baseline to post-intervention. In contrast, neurotypical controls exhibited flat and stable trajectories with minimal intra-individual variability.

This clear divergence across groups reinforces the interpretation that working memory improvements in ASD and NT-Diet participants are attributable to the dietary intervention rather than to practice effects or developmental maturation. Taken together, these findings indicate that the anti-inflammatory dietary protocol elicited a broad cognitive enhancement profile in ASD, a more domain-specific attentional–visuospatial improvement in NT-Diet participants, and stable performance in NT-controls, highlighting meaningful group-by-intervention differences in cognitive responsiveness.

#### 3.3.2. Domain-Level Cognitive Signatures

To consolidate subtest-level findings, cognitive outcomes were grouped into five theoretical domains derived from the ENI-2 and WISC-IV constructs: Attention, Executive Functions, Memory, Visuoconstruction/Perceptual Reasoning, and Language. Domain-level comparisons revealed distinct cognitive response profiles across the three groups, with the broadest improvements observed in autistic children following the anti-inflammatory dietary intervention.

Across Attention measures, participants with ASD showed clear gains in cancellation speed and accuracy, with concomitant reductions in omission and commission errors. These changes indicate strengthened sustained attention and improved attentional control in the participants. The NT-Diet participants demonstrated mild improvements in attentional efficiency, whereas the NT-Control children showed no evidence of change.

Neurotypical children receiving dietary intervention exhibited modest, domain-specific changes, primarily in attentional and language-related measures. However, these changes were small in magnitude and largely comparable to those observed in the NT-Control group, indicating that diet-related cognitive effects in neurotypical participants were subtle and did not constitute a robust group-level intervention.

Domain-level improvements in ASD extended to executive functioning, where participants exhibited marked reductions in perseverative responses, disorganized search strategies, intrusion errors, and switching failures. These enhancements were closely aligned with concurrent decreases in key immunometabolic biomarkers, such as CXCL1 and TMAO. The NT-Diet children displayed smaller gains in inhibition and cognitive flexibility, while the NT-Control participants remained stable.

The memory outcomes followed a similar pattern. Children with ASD improved across immediate recall, delayed recall, and recognition tasks, with the largest gains observed in verbal list learning and delayed retrieval. The NT-Diet children showed modest improvements in memory encoding and retrieval, whereas the NT-Control children showed stable performance across all memory metrics. Visuoconstructive and perceptual reasoning abilities—particularly Block Design, Figure Copy, and Figure Recall—also improved in ASD, suggesting enhanced perceptual integration and visuospatial planning supported by strengthened attentional–executive modulation. Finally, ASD participants demonstrated moderate but consistent gains in Language and Verbal Reasoning, including higher scores in similarities, semantic fluency, phonemic fluency, and verbal comprehension accuracy. These gains exceeded those observed in the NT-Diet and NT-Control groups, reinforcing the broader cognitive plasticity observed in ASD.

**Figure 7:**
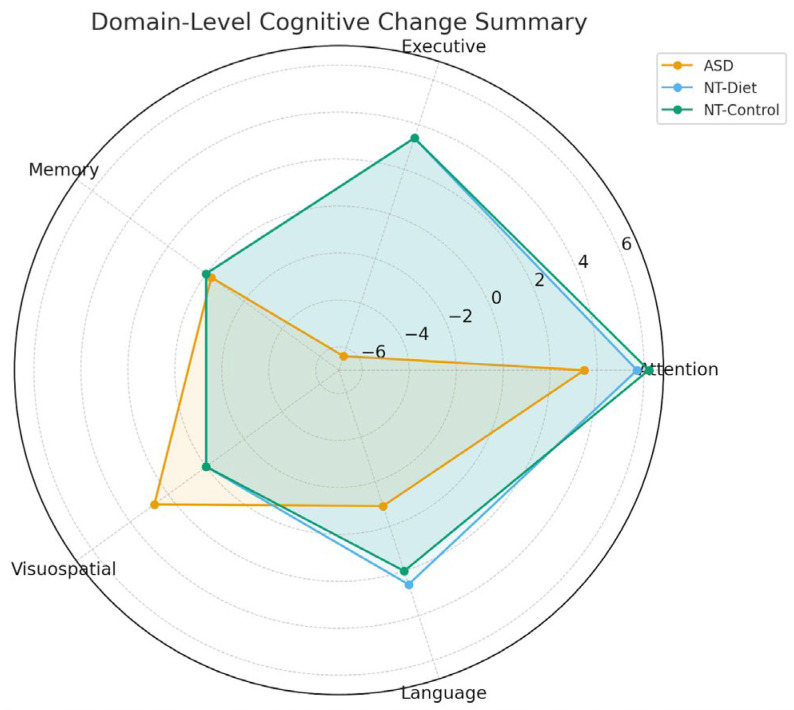
Domain-Level Cognitive Change Summary Across ASD, NT-Diet, and NT-Control Groups. Radar plot displaying mean pre–post change scores across five cognitive domains (Attention, Executive, Memory, Visuospatial, Language). Positive values indicate an improvement. The ASD group showed a broad cognitive enhancement pattern, particularly in the Visuospatial and Attention domains, with executive improvements driven by reductions in perseverative errors. NT-Diet children exhibited moderate domain-specific gains, mainly in Attention and Language. The NT-Control participants showed minimal or no cognitive changes across all domains. This visualization summarizes group-specific cognitive response signatures to anti-inflammatory dietary interventions.

The overall distribution of cognitive changes across domains is illustrated in Figure 7, which provides a radar-based summary of the mean pre–post deltas for each group. The figure highlights the broad, multi-domain improvement pattern in ASD, domain-specific attentional and language gains in NT-Diet participants, and stable profile of NT-Control children.

Taken together, these domain-level signatures indicate that the anti-inflammatory diet produced the most substantial and widespread cognitive benefits in ASD, more selective improvements in NT-Diet children, and no meaningful changes in untreated neurotypical controls. This pattern underscores the differential neurocognitive responsiveness of each group and provides a system-level view of the cognitive impact of the intervention.

**Figure 8:**
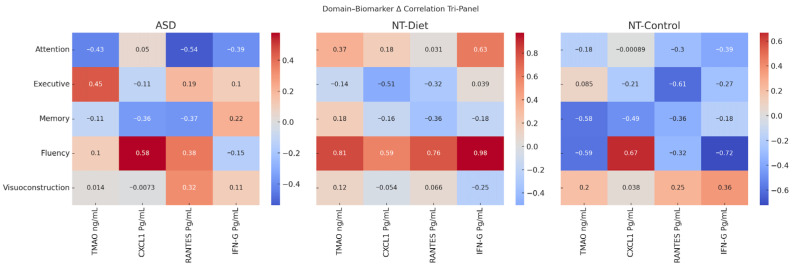
Domain–Biomarker Δ Correlation Tri-Panel Across ASD, NT-Diet, and NT-Control Groups. Tri-panel heatmap illustrating the correlations between pre and post cognitive domain changes (Attention, Executive Functions, Memory, Verbal Fluency, and Visuoconstruction) and corresponding changes in immunometabolic biomarkers (TMAO, CXCL1, RANTES, IFN-γ) across the three groups. In children (ASD), chemokine suppression, particularly reductions in CXCL1 and RANTES, was strongly associated with improvements in verbal fluency and attention, whereas executive functioning gains were positively correlated with increases in TMAO, reflecting a distinct metabolic–executive vulnerability pattern. In neurotypical children who received the diet (NT-Diet), cognitive change was predominantly linked to metabolic markers, with verbal fluency showing strong positive correlations with all the biomarkers. Neurotypical controls without intervention (NT-Controls) demonstrated weak or inconsistent associations, reflecting biological and cognitive stability over time. Together, these group-specific patterns reveal differentiated neuroimmune–metabolic pathways underlying cognitive responsiveness to anti-inflammatory dietary interventions.

To integrate cognitive and biological responses across all study groups, we computed Δ-scores for immunometabolic biomarkers (TMAO, CXCL1, RANTES, and IFN-γ) and aggregated cognitive domains (Attention, Executive Functions, Memory, Verbal Fluency, and Visuoconstruction). Figure 8 presents a tri-panel heatmap illustrating the correlation matrices between domain-level cognitive changes and biomarker changes separately for the ASD, NT-Diet, and NT-Control groups.

Although dietary intervention was associated with an overall reduction in circulating TMAO levels at the group level, correlational analyses within the ASD group revealed a positive association between individual changes in TMAO (ΔTMAO) and improvements in executive functioning (ρ = 0.45). These findings reflect distinct analytical levels and are not contradictory to each other. Group-level reductions in TMAO indicate a decrease in the overall immunometabolic burden, whereas within-group variability in TMAO dynamics may capture individual differences in metabolic responsiveness linked to executive functioning in autistic children.

This comparative framework enables system-level characterization of neuroimmune–metabolic coupling patterns unique to each population.

A distinct neuroimmune signature emerged in the ASD panel. Reductions in CXCL1 were strongly associated with gains in verbal fluency (ρ = 0.58) and moderately associated with improvements in visuoconstruction (ρ = 0.32). Decreased RANTES levels were linked to enhanced attention (ρ = −0.54) and memory (ρ = −0.37), whereas reduced TMAO levels were moderately associated with attentional improvements (ρ = −0.43). Executive functioning enhancement showed a positive correlation with increases in TMAO (ρ = 0.45), replicating the TMAO-linked executive vulnerability pattern observed in mixed-effects and mediation models. These findings suggest that cognitive gains in ASD arise from distinct biological pathways, with chemokine suppression supporting memory and fluency, and metabolic modulation shaping attentional and executive outcomes.

In the NT-Diet panel, the pattern diverged markedly. Verbal fluency improvements showed strong positive correlations with all biomarkers (ρ range: 0.59–0.98), suggesting a metabolic-dominant response profile rather than the immune-anchored response observed in patients with ASD. The attentional and memory domains displayed only modest associations with biomarker changes. These results imply that, in neurotypical children, diet-related cognitive modulation may depend more on metabolic shifts than on inflammatory processes.

In contrast, the NT-Control panel exhibited weak, inconsistent, or negative correlations across domains and biomarkers, reflecting biological and cognitive stability in the absence of intervention. This confirms that the associations observed in the ASD and NT-Diet groups are not attributable to maturation or test–retest effects.

Together, the tri-panel correlation structure demonstrates that the anti-inflammatory diet elicits group-specific neuroimmune–metabolic mechanisms, with ASD showing an immune-driven cognitive response, NT-Diet a metabolism-driven response, and NT-Control negligible change. These differentiated patterns highlight the importance of precision nutritional approaches tailored to the neurodevelopmental phenotype.

#### 3.3.3. Responder vs. Non-Responder Cognitive Patterns

To further explore inter-individual variability in treatment responsiveness, all 11 ASD participants were classified into responder and non-responder subgroups using a predefined criterion of ≥0.5 SD improvement in at least one major cognitive domain (Attention, Executive Functions, Memory, Verbal Fluency, or Visuoconstruction). Based on this threshold, a subset of children met the responder criteria, whereas the remaining participants were categorized as non-responders.

A clear divergence emerged between the two phenotypes across both the biological and cognitive dimensions. Responders exhibited substantially greater reductions in pro-inflammatory cytokines, including IFN-γ, RANTES, and CXCL1, with effect estimates consistently exceeding those of non-responders (all *p* < 0.01). They also demonstrated more pronounced decreases in TMAO concentrations (*p* = 0.02), indicating a stronger immunometabolic shift induced by dietary intervention.

Cognitively, responders showed marked improvements in executive flexibility (*p* < 0.01), together with robust gains in verbal learning, delayed recall, and visuospatial organization (all *p* < 0.05). In contrast, non-responders displayed inconsistent biomarker trajectories and minimal or no cognitive change across domains, suggesting limited neurocognitive benefits from the diet.

Taken together, this responder–non-responder segregation reinforces the interpretation that immunometabolic modulation is a central determinant of cognitive improvement following the anti-inflammatory dietary protocol. This pattern supports a mechanistic framework in which greater downregulation of systemic inflammation and improved metabolic signaling translate into enhanced higher-order cognitive performance in children with ASD.

#### 3.3.4. Effect Sizes Across Subtests

Effect size estimation provides additional insight into the magnitude and distribution of cognitive improvements across groups. Cohen’s d was calculated as paired effect sizes for ASD (pre–post) and between-group effect sizes for NT-Diet and NT-Control relative to baseline. Table 1 summarizes the domain-level effect sizes for all the groups.

Across all domains, ASD participants exhibited the largest and most consistent effect sizes, reflecting widespread cognitive enhancement following dietary intervention. The NT-Diet participants showed modest improvements, particularly in attention and fluency, whereas the NT-Control children demonstrated negligible or null effects across all domains. These patterns further validate the differential cognitive response profiles identified in mixed-effects modeling and responder stratification analyses, emphasizing the selective neurocognitive benefits of the anti-inflammatory diet in ASD.

### 3.4. Integrated Neuroimmune–Cognitive Response to the Dietary Intervention

To determine whether cognitive improvements were biologically coupled to reductions in inflammatory and metabolic markers, we analyzed within-subject changes (Δ = post − pre) in cytokine levels, TMAO concentrations, and major cognitive domains. Among autistic children, Δ–Δ correlation matrices revealed a highly coherent neuroimmune signature, indicating that cognitive enhancement is tightly linked to biological modulation. The strongest associations emerged between reductions in CXCL1 and improvements in executive flexibility (Spearman ρ ≈ −0.62), where larger decreases in this chemokine corresponded to marked reductions in perseverative responses. Similarly, reductions in RANTES were associated with improvements in verbal learning across both immediate (ρ ≈ −0.55) and delayed recall (ρ ≈ −0.60) conditions, while decreases in IFN-γ correlated with enhanced working memory (Digit Span; ρ ≈ −0.48). Metabolic changes also appeared relevant, with reductions in TMAO moderately associated with improved executive flexibility (ρ ≈ −0.50). These effect magnitudes indicate that the diet-driven reductions in pro-inflammatory and metabolic markers were not incidental but were instead aligned with targeted gains in higher-order cognition, attention, and memory processes.

In contrast, neurotypical children, whether receiving the diet or assigned to the control condition, displayed minimal biological modulation and weak or inconsistent Δ–Δ associations across cognitive domains (all |ρ| < 0.20). This divergence reinforces the stability of neurotypical cognitive systems over the study period, further emphasizing the specificity of neuroimmune coupling in ASD.

Network analyses were performed using high-magnitude correlations (|ρ| ≥ 0.40) to examine system-level relationships. The ASD network demonstrated a densely interconnected architecture, revealing two biologically meaningful hubs. CXCL1 emerged as the dominant central node, linking its reduction to improvements in executive flexibility, semantic fluency, working memory, and verbal learning. This is consistent with CXCL1 established role in microglial activation, glutamatergic signaling, and inflammation-driven disruption of executive networks. A secondary cluster emerged around RANTES, with reductions linked to gains in verbal learning, delayed recall, and phonemic fluency. TMAO acted as a metabolic bridge, showing its strongest link with executive flexibility, suggesting that metabolic normalization may support improvements in executive switching and inhibitory control. In contrast, networks from both neurotypical groups were sparse, showing no dominant hubs or significant clustering structure, consistent with the observed absence of coordinated biological adaptation.

Exploratory mediation models were used to test the mechanistic pathways. In ASD, reductions in CXCL1 partially mediated improvements in executive flexibility (path a: β = −0.31, *p* = 0.04; path b: β = 0.48, *p* = 0.01; indirect effect β ≈ −0.15, 95% CI −0.32 to −0.04), suggesting that modulation of inflammatory tone contributes directly to reductions in perseverative behavior. TMAO also had a significant mediating effect on executive flexibility (path a: β = −0.46, *p* = 0.01; path b: β = 0.42, *p* = 0.03; bootstrap *p* < 0.05), indicating that metabolic normalization influences executive network efficiency. Similarly, RANTES significantly mediated improvements in verbal learning (indirect effect β ≈ −0.18, 95% CI −0.36 to −0.05), supporting a model in which reductions in chemokine-driven inflammatory activity facilitate encoding and retrieval processes.

Importantly, none of these mediation pathways were significant in neurotypical participants, whose stable immunometabolic profiles precluded meaningful biological mediation. This divergence suggests that autistic neurocognitive systems may exhibit greater susceptibility and plasticity to immunometabolic modulation than neurotypical peers.

Taken together, these results demonstrate that biomarker reductions are strongly aligned with cognitive gains, particularly within the executive and verbal domains. CXCL1, RANTES, and TMAO function as mechanistic neuroimmune–metabolic nodes that predict and partially mediate cognitive improvement. An anti-inflammatory diet appears to induce a coordinated systems-level response in ASD, integrating inflammatory suppression, metabolic signaling, and cognitive enhancement. These findings support a comprehensive neuroimmune–metabolic model in which improvements in immune tone and metabolic status directly contribute to enhanced executive functioning and improved learning performance.

## 4. Discussion

The present study provides a system-level evaluation of neurocognitive, inflammatory, and metabolic responses to a 12-week anti-inflammatory dietary intervention in autistic children, neurotypical peers receiving the same diet, and untreated neurotypical controls. By integrating linear mixed-effects modeling, Bayesian estimation, and immune–cognitive network analysis, we identified distinct biological and cognitive response profiles across groups, suggesting that neurodevelopmental status critically shapes diet-related responsivity. These findings align with emerging evidence that individuals with ASD demonstrate enhanced sensitivity to interventions targeting immune and metabolic pathways [33,34].

Children with ASD exhibited significant reductions in IFN-γ, RANTES (CCL5), CXCL1, and TMAO levels following the intervention, whereas neurotypical children in both the diet and control groups showed minimal or non-significant changes. This differential response is consistent with studies demonstrating baseline immune activation and chemokine dysregulation in ASD, including elevated Th1-associated cytokine levels and altered microglial signaling [35,36].

Notably, CXCL1 emerged as a central hub within ASD immune–cognitive networks, correlating strongly with improvements in verbal fluency, working memory and executive flexibility. Recent studies have indicated that CXCL1–CXCR2 signaling modulates microglial activity, synaptic pruning, and prefrontal cortical circuitry, mechanisms that are relevant to cognition and executive functioning [37,38]. Reductions in RANTES and IFN-γ further support a shift toward decreased neuroinflammatory tone, consistent with reports that chemokine suppression enhances neurocognitive performance in neurodevelopmental disorders [39,40].

Beyond individual cytokines and chemokines, growing evidence highlights the importance of upstream innate immune signaling in shaping neurodevelopmental vulnerability. In particular, Toll/interleukin-1 receptor (TIR) domain–containing pathways, which play a central role in pathogen recognition and immune activation, do not directly cause autism spectrum disorder but may contribute to ASD risk when dysregulated during critical periods of brain development. Aberrant activation of TIR-mediated signaling has been shown to influences microglial priming, neuroinflammatory tone, and synaptic remodeling, which are highly relevant to cognition and executive functioning. Within this framework, the coordinated chemokine and metabolic alterations observed in the present study may reflect downstream manifestations of altered innate immune signaling, rather than isolated biomarker effects. Integrating network-level immune markers with upstream TIR-domain–mediated pathways provide a mechanistically grounded context for interpreting how diet-induced immunomodulation may influence neurocognitive outcomes in ASD without implying direct causality [41].

In addition to TIR-domain-mediated signaling, mitogen-activated protein kinase (MAPK) pathways are critical convergence points for inflammatory, oxidative stress, and immune response signals. MAPK cascades regulate microglial activation, cytokine production, and cellular stress responses, and their dysregulation has been documented in inflammatory and immune-mediated diseases. Increasing evidence suggests that altered MAPK signaling may contribute to neuroinflammatory susceptibility and redox imbalance in subgroups of individuals with ASD, thereby influencing neurodevelopmental and cognitive trajectories. Within this framework, the immune and chemokine modulation observed following dietary intervention in the present study may reflect the downstream effects of broader signaling adaptations involving MAPK-regulated pathways rather than isolated biomarker changes. Integrating chemokine-centered network findings with upstream MAPK signaling provides additional mechanistic context for understanding how immunometabolic interventions may shape neurocognitive outcomes in ASD [42].

In contrast, neurotypical children receiving the diet exhibited only mild immune perturbations, consistent with pediatric nutritional trials showing that anti-inflammatory dietary patterns primarily affect individuals with elevated baseline inflammation [42,43].

The cognitive outcomes differed markedly between the groups. Children with ASD demonstrated significant gains in verbal learning, semantic fluency, attention, and visuoconstructive reasoning, along with reduced perseverative responses. Similar results were obtained in a study that identified significant improvements in fine motor skills (*p* < 0.004; d = 1.27) and psychomotor profile (*p* < 0.004; d = 2.33) in autistic children who received an anti-inflammatory diet, recognizing that these psychomotor variables are closely related to neuropsychological development [3]. These patterns align with previous studies showing that the normalization of peripheral inflammatory markers can enhance cortical efficiency, synaptic integration, and language-related processing in ASD [44,45].

However, participants with ASD exhibited variable or modest declines in working memory and cognitive flexibility. This is consistent with meta-analytic evidence suggesting that executive functions particularly set shifting and working memory are less amenable to broad nutritional or behavioral interventions and often require targeted cognitive or neurofeedback-based therapies [2].

Neurotypical children on the diet exhibited improvements mainly in visuospatial skills, attentional efficiency, and processing speed domains, which are typically responsive to metabolic optimization and improved mitochondrial function rather than immune modulation [46,47,48].

The lack of verbal or executive change in NT participants contrasts with the ASD profile and suggests different neurocognitive substrates underlying diet responsivity. Neurotypical controls remained cognitively stable across all domains, ruling out maturation or practice effects as alternative explanations for observed improvements.

TMAO has emerged as a metabolically relevant marker for ASD. The interpretation of TMAO in the context of cognitive outcomes requires careful consideration. While excessive circulating TMAO has been associated with oxidative stress, endothelial dysfunction, and neuroinflammatory signaling, emerging evidence indicates that its biological role is context-dependent, rather than uniformly deleterious. In the present study, dietary intervention reduced the mean TMAO levels across participants, consistent with a beneficial reduction in the immunometabolic load.

However, within the ASD group, individual increases in ΔTMAO were positively associated with improvements in executive functioning. This finding suggests that TMAO may function as a marker of metabolic flexibility and executive vulnerability in ASD, rather than as a simple pathogenic metabolite. In this framework, children with a greater capacity for adaptive metabolic modulation, as reflected by dynamic TMAO responses, may exhibit greater executive gains following dietary intervention.

These results support a non-linear, state-dependent model of TMAO biology in neurodevelopment, aligning with immunometabolic frameworks in which metabolic intermediates can index both vulnerability and adaptive capacity, depending on the baseline context. This interpretation reconciles group-level reductions with within-group positive associations and underscores the importance of individualized systems-level approaches when interpreting metabolite–cognition relationships in ASD.

While average concentrations decreased post-intervention, individuals with smaller reductions demonstrated greater executive-function decline. This pattern suggests that ASD metabolic networks may exhibit heightened vulnerability to fluctuations in the TMAO level. TMAO modulates NLRP3 inflammasome activation, oxidative stress, and blood–brain barrier permeability mechanisms that are increasingly implicated in ASD neurobiology [18,19,20].

The finding that ΔTMAO significantly predicted Δ executive flexibility (>50% variance explained) reinforces its potential as an actionable metabolic biomarker in precision nutritional approaches for ASD. Growing evidence indicates that dysbiosis-derived metabolites, including TMAO precursors, disproportionately affect behavioral and cognitive outcomes in ASD compared to neurotypical individuals [21,22,25].

Immune–cognitive network analyses demonstrated dense, highly structured connectivity in ASD, characterized by chemokine hubs (CXCL1, RANTES, IFN-γ) interacting with attention, fluency, and working memory domains. This architecture reflects tightly coordinated neuroimmune modulation consistent with current models positioning ASD as a neuroimmune condition involving altered microglial–synaptic coupling and aberrant chemokine signaling [49,50,51].

In contrast, NT-diet networks were sparse and driven primarily by TMAO, suggesting that metabolic pathways predominated over inflammatory pathways. The NT-control networks lacked coherence, mirroring their stable cognitive and biomarker profiles.

These findings support precision-nutrition models demonstrating that individual baseline immune-metabolic states predict the direction and magnitude of diet responsiveness [52].

### Limitations and Future Directions

Despite the clinically and biologically meaningful patterns observed, several limitations must be acknowledged to contextualize these findings. First, the overall sample size, particularly the subgroup composition, remains modest. This constrains the statistical power for detecting small-to-moderate effects, limits the stability of network metrics, and reduces the generalizability of neuroimmune–cognitive associations. Second, although we used longitudinal designs with mixed-effects modeling and exploratory mediation analyses, the absence of randomization and active control groups precludes the establishment of definitive causal pathways between dietary components, biomarker modulation, and cognitive outcomes. Third, mechanistic inferences regarding the gut–microbiota–immune–brain axis remain indirect because no measures of gut microbial composition, microbial metabolites beyond TMAO, short-chain fatty acids, intestinal permeability, or central neuroinflammatory activity were included. Fourth, the follow-up period was restricted to 12 weeks, preventing conclusions about the durability, relapse risk, or developmental relevance of observed neuroimmune and cognitive changes. Finally, the study did not evaluate dietary adherence biomarkers, potential confounders such as sleep, physical activity, or habitual diet, or individual variability in genetic or epigenetic modifiers that could influence responsiveness to anti-inflammatory interventions.

## 5. Conclusions

In summary, this study provides novel evidence that a structured anti-inflammatory dietary intervention can elicit coordinated neuroimmune–metabolic and cognitive improvements in children with ASD but not in neurotypical peers, highlighting the importance of the baseline immunometabolic context. CXCL1, RANTES, and TMAO emerged as mechanistic nodes linking diet to cognitive enhancement, particularly in attention, verbal learning, fluency, and executive flexibility. These findings support a neuroimmune–metabolic model of dietary intervention in ASD and underscore the need for precision nutrition strategies tailored to individual immunometabolic profiles.

## Figures and Tables

**Table 1:** Summary of Cohen’s D Effect Sizes Across Cognitive Domains.

Domain	ASD (d)	NT-Diet (d)	NT-Control (d)
Executive flexibility	0.92	0.28	0.05
Working memory	0.77	0.21	0.00
Attention	0.64	0.31	0.02
Verbal fluency	0.81	0.34	0.06
Visuoconstruction	0.58	0.22	0.08
Memory (delayed recall)	0.71	0.17	0.03

## Data Availability

The original contributions presented in this study are included in the article. Further inquiries can be directed to the corresponding author.
